# Supplementary material for: Resolving the full spectrum of human genome variation using Linked-Reads
Source: Genome Res. 2019 Apr;29(4):635–45. doi: 10.1101/gr.234443.118 (PMC6442396; doi:10.1101/gr.234443.118)
Supplement: Supplemental Material [file supp_gr.234443.118_Supplemental_longranger-2.2.zip › longranger-2.2.tar.bz2!/lr2.2-public/lib/stan/cmdstan/stan/doc/api/html/agrad_2rev_2functions_2owens__t_8hpp_source.html]

Stan: src/stan/agrad/rev/functions/owens\_t.hpp Source File


|  |  |
| --- | --- |
|  | Stan  2.5.0  probability, sampling & optimization |


- Main Page
- Related Pages
- Namespaces
- Classes
- Files

- File List
- File Members

All Classes Namespaces Files Functions Variables Typedefs Enumerations Enumerator Friends Macros Pages

- src
- stan
- agrad
- rev
- functions

owens\_t.hpp

Go to the documentation of this file.

1 #ifndef STAN\_\_AGRAD\_\_REV\_\_FUNCTIONS\_\_OWENS\_\_T\_HPP

2 #define STAN\_\_AGRAD\_\_REV\_\_FUNCTIONS\_\_OWENS\_\_T\_HPP

3

4 #include <math.h>

5 #include <stan/agrad/rev/var.hpp>

6 #include <stan/agrad/rev/internal/vv\_vari.hpp>

7 #include <stan/agrad/rev/internal/vd\_vari.hpp>

8 #include <stan/agrad/rev/internal/dv\_vari.hpp>

9 #include <stan/math/constants.hpp>

10 #include <stan/math/functions/square.hpp>

11 #include <boost/math/special\_functions/owens\_t.hpp>

12

13 namespace stan {

14  namespace agrad {

15

16  namespace {

17

18  using stan::math::pi;

19  class owens\_t\_vv\_vari : public op\_vv\_vari {

20  public:

21  owens\_t\_vv\_vari(vari\* avi, vari\* bvi) :

22  op\_vv\_vari(boost::math::owens\_t(avi->val\_, bvi->val\_), avi, bvi) {

23  }

24  void chain() {

25  using stan::math::INV\_SQRT\_TWO\_PI;

26  using stan::math::INV\_SQRT\_2;

27  const double neg\_avi\_sq\_div\_2 = -stan::math::square(avi\_->val\_) \* 0.5;

28  const double one\_p\_bvi\_sq = 1.0 + stan::math::square(bvi\_->val\_);

29

30  avi\_->adj\_ += adj\_ \* ::erf(bvi\_->val\_ \* avi\_->val\_ \* INV\_SQRT\_2)

31  \* std::exp(neg\_avi\_sq\_div\_2) \* INV\_SQRT\_TWO\_PI \* -0.5;

32  bvi\_->adj\_ += adj\_ \* std::exp(neg\_avi\_sq\_div\_2 \* one\_p\_bvi\_sq)

33  / (one\_p\_bvi\_sq \* 2.0 \* pi());

34  }

35  };

36

37  class owens\_t\_vd\_vari : public op\_vd\_vari {

38  public:

39  owens\_t\_vd\_vari(vari\* avi, double b) :

40  op\_vd\_vari(boost::math::owens\_t(avi->val\_, b), avi, b) {

41  }

42  void chain() {

43  using stan::math::INV\_SQRT\_TWO\_PI;

44  using stan::math::INV\_SQRT\_2;

45  using stan::math::square;

46

47  avi\_->adj\_ += adj\_ \* ::erf(bd\_ \* avi\_->val\_ \* INV\_SQRT\_2)

48  \* std::exp(-square(avi\_->val\_) \* 0.5)

49  \* INV\_SQRT\_TWO\_PI \* -0.5;

50  }

51  };

52

53  class owens\_t\_dv\_vari : public op\_dv\_vari {

54  public:

55  owens\_t\_dv\_vari(double a, vari\* bvi) :

56  op\_dv\_vari(boost::math::owens\_t(a, bvi->val\_), a, bvi) {

57  }

58  void chain() {

59  using stan::math::INV\_SQRT\_2;

60  using stan::math::INV\_SQRT\_TWO\_PI;

61  using stan::math::square;

62  const double one\_p\_bvi\_sq = 1.0 + stan::math::square(bvi\_->val\_);

63

64  bvi\_->adj\_ += adj\_ \* std::exp(-0.5 \* square(ad\_)

65  \* one\_p\_bvi\_sq)

66  / (one\_p\_bvi\_sq \* 2.0 \* pi());

67  }

68  };

69  }

70

82  inline var owens\_t(const var& h,

83  const var& a) {

84  return var(new owens\_t\_vv\_vari(h.vi\_, a.vi\_));

85  }

86

98  inline var owens\_t(const var& h,

99  const double& a) {

100  return var(new owens\_t\_vd\_vari(h.vi\_, a));

101  }

102

114  inline var owens\_t(const double& h,

115  const var& a) {

116  return var(new owens\_t\_dv\_vari(h, a.vi\_));

117  }

118

119  }

120 }

121 #endif

stan::math::square

T square(const T x)

Return the square of the specified argument.

**Definition:** square.hpp:22

stan::math::INV\_SQRT\_TWO\_PI

const double INV\_SQRT\_TWO\_PI

**Definition:** constants.hpp:150

dv\_vari.hpp

stan::agrad::erf

fvar< T > erf(const fvar< T > &x)

**Definition:** erf.hpp:17

square.hpp

stan::agrad::square

fvar< T > square(const fvar< T > &x)

**Definition:** square.hpp:15

vd\_vari.hpp

stan::agrad::var::vi\_

vari \* vi\_

Pointer to the implementation of this variable.

**Definition:** var.hpp:40

stan::agrad::owens\_t

fvar< T > owens\_t(const fvar< T > &x1, const fvar< T > &x2)

**Definition:** owens\_t.hpp:17

stan::math::INV\_SQRT\_2

const double INV\_SQRT\_2

The value of 1 over the square root of 2, .

**Definition:** constants.hpp:26

var.hpp

stan::agrad::var

Independent (input) and dependent (output) variables for gradients.

**Definition:** var.hpp:27

vv\_vari.hpp

stan::math::pi

double pi()

Return the value of pi.

**Definition:** constants.hpp:77

constants.hpp

stan::agrad::exp

fvar< T > exp(const fvar< T > &x)

**Definition:** exp.hpp:16


---

[ Stan Home Page ]

*© 2011–2014,
Stan Development Team.*
